# Effect of Electronic Acceptor Segments on Photophysical Properties of Low-Band-Gap Ambipolar Polymers

**DOI:** 10.1155/2013/890215

**Published:** 2013-01-10

**Authors:** Yuanzuo Li, Jingang Cui, Jianing Zhao, Jinglin Liu, Peng Song, Fengcai Ma

**Affiliations:** ^1^College of Science, Northeast Forestry University, Harbin 150040, China; ^2^School of Physics and Optoelectronic Technology, Dalian University of Technology, Dalian 116024, China; ^3^College of Science, Jiamusi University, Jiamusi 154001, China; ^4^Department of Physics, Liaoning University, Shenyang 110036, China

## Abstract

Stimulated by a recent experimental report, charge transfer and photophysical properties of donor-acceptor ambipolar polymer were studied with the quantum chemistry calculation and the developed 3D charge difference density method. The effects of electronic acceptor strength on the structure, energy levels, electron density distribution, ionization potentials, and electron affinities were also obtained to estimate the transporting ability of hole and electron. With the developed 3D charge difference density, one visualizes the charge transfer process, distinguishes the role of molecular units, and finds the relationship between the role of DPP and excitation energy for the three polymers during photo-excitation.

## 1. Introduction 

Ambipolar devices are very attractive organic semiconductors due to their high chemical stability and uncommon versatility; particularly, ambipolar copolymer films have been applied as active materials in the field of organic optoelectronic materials [1–6]. For example, ambipolar organic field-effect transistors, which are capable of both p- and n-channel operations by changing the polarity of the gate voltage, are gaining attention as an alternative approach to mimicking complementary metal-oxide semiconductor (CMOS) digital integrated circuits for achieving high-performance and cost-effective circuits in organic electronics. On one hand, the semiconductors suitable as single component channel materials should facilitate the formation of an exciton through the cation and anion radicals, and they show both stable hole and electron transporting characteristics; on the other hand, the semiconductors are required to have a well match between ambipolar structures and metal electrodes to balance the charge injection barriers between the relative positions of HOMO and LUMO energy levels and the work function of electrodes.

The 1, 4-diketo-3, 6-diarylpyrrolo [3, 4-c] pyrroles diketopyrrolopyrroles (DPPs) are chromophoric systems that combine in a rigid planar structural frame. Generally, the DPP unit possesses the strong electron-withdrawing capability. When combining with appropriate building blocks (such as thiophene, phenylene, and benzofuran building blocks), the certain DPP-containing polymers show ambipolar characteristic. Meanwhile, this structural adjustment should result in tunable band gap, energy levels, and molecular packing. Therefore, DPP-containing polymers show wide application prospect in the field of organic photovoltaics [7, 8], electroluminescence device [9, 10], organic field-effect transistors [11, 12], and logic circuits [13, 14]. Recently, electronic, physical, and transistor properties of a family of donor-acceptor polymers (which consist of DPP coupled with neutral benzene (B), the weakly accepting benzothiadiazole (BT) and the strongly accepting benzobisthiadiazole (BBT)) have been experimented [15]. The report found that the rational design can realize the conversion from unipolar to ambipolar donor-acceptor polymer. Although experimental study made tremendous progress, it is important to understand the relationship between chemical structures and the optical and electronic properties of the organic ambipolar layer from the molecular level. Theoretical calculations not only provide some parameters (such as electron transition, ionization potential (IP), and electron affinity (EA)) affecting the optical response and charge transport ability, but also reveal the microscopic mechanism behind the experiment and give theoretical basis to rational design of new functional materials. In this paper, we attempt to study the electronic structure and photo-physical nature from the viewpoint of theory, to deeply insight into photo-induced charge transfer process with the developed 3D cube representations [16–19]. This method can visually distinguish the role of molecular units during the photo-excitation. The article is organized as follows: firstly, the ground and excited state geometries are optimized with density functional theory. Secondly, molecular orbital shape and energy, IP and EA are estimated. Lastly, charge transfer and excited states properties are studied, and role of molecular units upon excitation is distinguished by the visualized analysis. 

## 2. Methods

**Figure 1:**
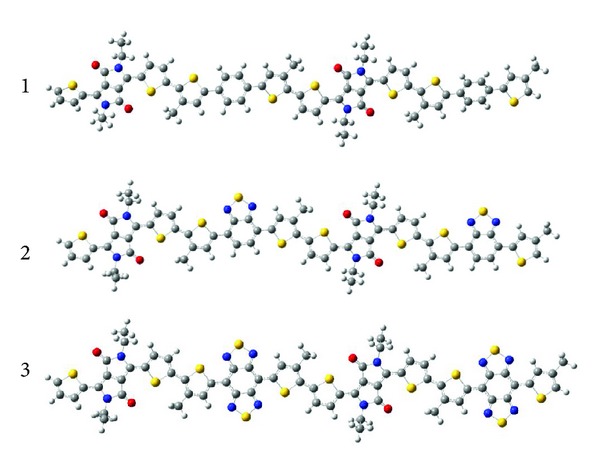
Molecular structures of PBDPP, PBTDPP, and PBBTDPP.

The ground state geometries of PBDPP, PBTDPP, and PBBTDPP (see Figure 1) were optimized with density functional theory (DFT) [20], B3LYP functional [21–23], and 6-31G (d) basis set. The alkyl groups on five members were replaced with methoxyl groups due to the fact that the alkyl groups do not significantly affect the equilibrium and optical property of fluorene-based polymer [24]. The excited-state geometries were optimized by time-dependent DFT (TD-DFT) [25], by using the 6-31G (d) basis set. The electronic transition of them for absorption and fluorescence was calculated at both ground-state and excited-state optimized geometries by means of TD-DFT, Cam-B3LYP functional [26] with the same basis set. The photoinduced intramolecular charge transfer process was visualized with the 3D cube representations. 3D charge difference density indicates that the electronic redistribution involving the whole structure takes place upon excitation [16, 17, 27, 28]. The charge difference density is defined as

(1) Δρuu(r⃑)=∑a∈unocci,j=occCuajCuaiφj(r⃑)φi(r⃑)−∑a,b∈unocci∈occCubiCuaiφb(r⃑)φa(r⃑),

where *C*_*uaj*_ is the *u*th eigenvector of the single configuration interaction (CI) Hamiltonian in the basis of the occupied Hartree-Fock molecular orbital *φ*_*i*_ and the unoccupied *φ*_*a*_ orbital [16, 27], and in this equation the first and the second terms stand for hole and electron, respectively. All the quantum chemical calculations were done with Gaussian 09 software [29]. The optical absorption spectra of them were done with GaussSum 2.1 [30].

## 3. Results and Discussion

### 3.1. Ground Optimization

The ground state structures of three molecular structures (PBDPP, PBTDPP, and PBBTDPP) have been optimized, and selected optimized bond length and dihedral angles of them were listed in Table 1. Comparing the dihedral angles of three polymers, it found that PBDPP, PBTDPP, and PBBTDPP displayed an obvious twisted configuration along the main molecular skeleton. For example, thiophene-diketopyrrolopyrrole (T-DPP) of PBDPP, PBTDPP, and PBBTDPP are 13.76026, 11.78848, and 10.44882 degree, respectively. The dihedral angles of thiophene-benzene (T-B) in PBDPP, thiophene-benzothiadiazole (T-BT) in PBTDPP, and thiophene-benzobisthiadiazole (T-BBT) are 21.51214, −1.82834, and −0.27066 degree, respectively. Although the PBBTDPP also has a certain twisted structure, it exhibits the well conplanar structure than the other two. The order of coplanarity is increased from PBDPP, PBTDPP to PBBTDPP. The date of intermolecular bond length demonstrated the conjugated degree of PBDPP is lower than that of PBTDPP and PBBTDPP. Therefore, introduction of BT and BBT improves the molecular coplanarity, which will lead to enhancement of electronic delocalization and improvement of their optical response.

**Figure 2:**
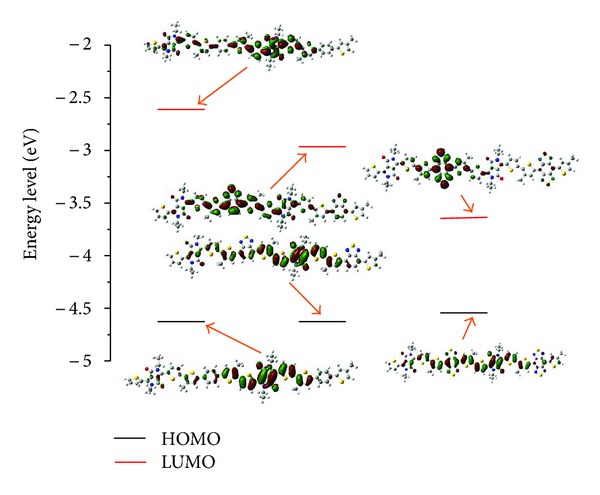
The energy levels and the electron density distribution of molecular orbitals.

It will be important to analyse the energy levels and the electron density distribution of molecular orbitals (MOs), since the change of electron density of MOs upon photo-excitation provides a reasonable qualitative indication of the excitation properties [31]. The energy levels of three polymers are indicated in Figure 2, and contour plot surfaces of HOMO and LUMO of them are inserted in Figure 2. As shown, for PBDPP electron densities of HOMO are mainly located in the two thiophens of first monomer, the DPP and two thiophens of second monomer. As compared with HOMO, the LUMO of PBDPP also resides the right two thiophens and the DPP and two thiophens, and a few of electron density is in the backbone chain of first monomer. For PBTDPP, electron density of the HOMO mainly resides in the whole dimer and electron density of the LUMO in the first monomer. Upon photo-excitation, there is a decreasing trend of electron density in the second monomer, and an increasing trend of electron density occurs in the BT unit of first monomer. The introduction of BT not only induces the change of electron density distribution, but also destabilizes the energy of LUMO and stabilizes the energy of HOMO, so PBTDPP has a lower energy gap than PBDPP. Among the three molecules, PBBTDPP has narrowest energy gap about 0.89 eV closed to the experiment value of 0.65 eV [15]. In addition, from the electron density distribution, it found that electron density of HOMO distributes over the whole backbone, while electron density of LUMO is mainly located in the BBT unit of the first monomer. So the character of MOs for PBBTDPP displays an obvious electron moving from the second monomer to the BBT unit.

Ionization potentials (IPs) and electron affinities (EAs) as the criterion of evaluating the injection ability of the hole and electron have been obtained by calculating adiabatic potential on the basis of neutral and charged optimization. For the three polymers, the value of ionization potentials for PBDPP, PBTDPP, and PBBTDPP is 5.259, 5.214, and 5.032 eV, respectively, that is, it decreases in this order PBBTDPP < PBTDPP < PBDPP; this means that for PBBTDPP with the lowest IP the hole injection is more easily than the others. Comparing the electron affinities of PBDPP, PBTDPP, and PBBTDPP, one found that they have an increasing trend, that is, EA value is 2.023 (PBDPP), 2.367 (PBTDPP), and 3.103 eV (PBBTDPP), respectively; so for the PBBTDPP the electron injection from the cathode to the electron transporting layer is more easily than the others.

### 3.2. Absorption Spectra and Charge Transfer Process

**Figure 3:**
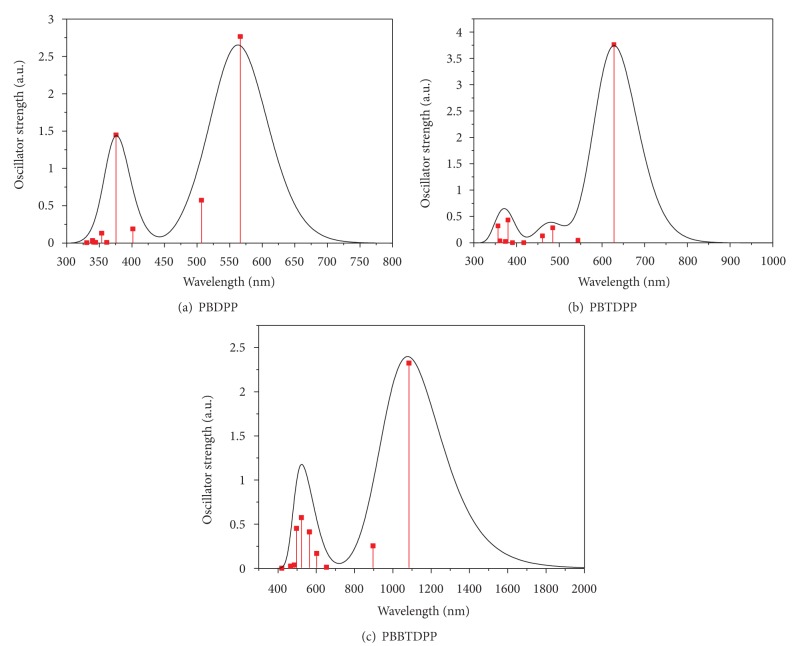
Absorption spectra of PBDPP, PBTDPP, and PBBTDPP.

Simulated absorption spectra of PBDPP, PBTDPP, and PBBTDPP are shown in Figure 3, and transition energies and the oscillator strengths are listed in Table 2. It is clearly seen from Figures 3(a) and 3(b) that the absorption peaks of PBDPP and PBTDPP are mainly on the range of visible and ultraviolet light, and the absorption peak is red shifted ~60 nm when the B is replaced by BT. By comparing Figures 3(a) and 3(c), one can see that shape of absorption spectra of PBBTDPP does not change, but the lowest strong absorption peak takes place further significantly red-shifted to the infrared region (to ~1084 nm). For the three polymers, the strongest absorption all corresponding to the S_1_ excited state that is composed of electron transition HOMO→LUMO (H→L). As shown in Table 2, for PBDPP the second absorption peak is at 375.63 nm, which is close to the second absorption peak of PBTDPP; but for PBBTDPP, the second absorption peak make red-shifted to be 522.83 nm.

**Figure 4:**
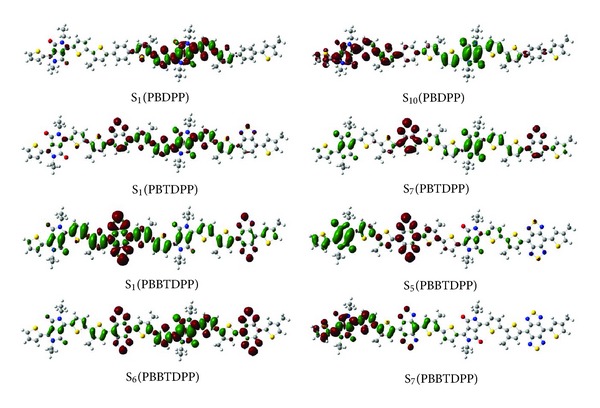
Charge different density of PBDPP, PBTDPP, and PBBTDPP for absorption (where red and green stand for electron and hole, resp.).

In order to understand the nature of absorption spectra, we study the change of charge density during excitation for the calculated ten excited states, and photoinduced intramolecular charge transfer in PBDPP, PBTDPP, and PBBTDPP on electronic transitions can be seen from Figure 4. For the S_1_ of PBDPP, electron and hole mainly in turn cover the two thiophens and the DPP and two thiophens of second monomer, and hence this state is characterized as *π*→π* electron transition. Simultaneously, we further checked the other states (see supporting material available online at http://dx.doi.org/10.1155/2013/890215). For the states (S_2_, S_3_, and S_4_) electron and hole are in turn distributed in the one monomer or the two monomers with the character of *π*→π* electron transition.

While for S_5_–S_10_ states closed to the different excitation energies, they have different charge transfer characters. For example, for S_6_ state electron move to DPP units and hole in the thiophens and benzenes, so in this state DPP serves as electron acceptor; for the S_7_–S_9_ states, DPP serves as electron donor owing to the more electron moving from it to the adjacent thiophens and benzenes. It is worth noting that for the S_10_ state, it is an obvious intramolecular charge transfer excited state, where electron moves from the DPP of right monomer to the DPP of left monomer, and DPP simultaneously serves as electron donor and acceptor during excitation. So it can be found that for PBDPP (containing neutral benzene (B)) the providing and accepting electronic rules of DPP have the characteristics of wavelength dependence. 

For PBTDPP, charge different density (see Figure 4) shows that photo-induced red electron is transferred to the BT subunit (where BT serves as electron acceptor), so this state is an intramolecular charge transfer state (ICT). While for the second absorption peak (S_7_), excitation takes place on the whole segments of PBTDPP. It is found in Figure 4 that red electron moves to two BT units and green hole in DPP units, so BT unit and DPP unit act as the electronic acceptor and donor group, respectively. 

For the PBBTDPP, the S_1_ state is an ICT state, where electron is transferred from DPP to BBT; therefore, for this strong absorption peak DPP serves as electronic donor and BBT as acceptor. Moreover, there are three excited states with closing oscillator strength, that is, S_5_ state (564 nm, *f* = 0.4115), S_6_ state (522.83 nm, *f* = 0.5742), and S_7_ state (497 nm, *f* = 0.4517), but they have different charge transfer characters. As shown in Figure 4, for S_5_ state red electron moves to the BBT unit and neighboring thiophen units of left monomer, and green hole resides in the two DPP units and neighboring thiophen units of two monomers, so this state is also ICT state. Exaction of S_6_ state also occurs on the leading backbone, where more electrons have arisen in the two BBT units. Different with the above-excited states, S_7_ state comes from the excitation of the single monomer, and electron moves from right thiophen unit of left monomer to the left thiophen unit. Then one can conclude that the second absorption band of PBBTDPP, including the S_5_, S_6_, and S_7_ states, strongly depends on the different ICT modes. Furthermore, for the above three states, BBT units all serve as electron acceptor displaying the strong ability of accepting electron. 

We therefore studied in detail the charge transfer of three polymers and visualized the function of every unit in photo-induction. When comparing the ICT states of three polymers, it is found that for the different excited states corresponding to different excitations, DPP, of PBDPP, PBTDPP, and PBBTDPP exhibits different abilities of electronic donor and acceptor gain or loss. Along with the increasing strength of donor groups (from the neutral benzene (B) to the strongly accepting benzobisthiadiazole (BBT)), the rules of electronic donor or acceptor of DPP for three polymers have a change from the initial wavelength dependence to the final accepting groups dependence. Especially for PBBTDPP, DPP nearly acts as the donor segment providing electron and their spectral region cover from 420 nm to 1084 nm.

### 3.3. Excited State Optimization and Fluorescence Properties

The geometries parameters of excited state optimization by using TDDFT method were listed in Table 1. It can be seen from Table 1 that the bond lengths of adjacent units for the excited state geometries have been mildly shorten about 0.02 angstrom (A) compared with the corresponding bond lengths of ground state geometries. However, the large changes calculated with TDDFT method have taken place in the dihedral angles of adjacent units. For example, for ground state the dihedral angle of T-DPP is 18.96641 (for PBDPP), 19.37309 (for PBTDPP), and 18.69428 (for PBBTDPP); and for excited state it is 18.09190 (for PBDPP), 18.40059 (for PBTDPP), and 17.93294 (for PBBTDPP), respectively. For T-B (for PBDPP), T-BT (for PBTDPP), and T-BBT (for PBBTDPP), the dihedral angles are changed from 21.51214, −1.82834, and −0.27066 to 1.39836, −0.47006, and −0.08738. Therefore, excited state geometries of all oligomers are more coplanar in comparison with ground-state geometries.

The fluorescence energies of all oligomers were calculated on the basis of excited state optimization, as shown in Table 3, and fluorescence lifetime was calculated with the Einstein transition probabilities [32] as follows:

(2) $$\begin{array}{c} \tau =\frac{c^{3}}{2{\left(E_{\text{Flu}}\right)}^{2}f}, \end{array}$$

where, *C*, *E*_Flu_, and *f* stand for the velocity of light, fluorescence energy, and oscillator strength, respectively. As shown in Table 3, fluorescence peaks of PBDPP and PBTDPP are 660.93 nm and 724.45 nm. Compared with the two formers, fluorescence peaks of PBBTDPP make red-shifted into the infrared region, and the oscillator strength of PBBTDPP is smaller than the others, which result in the longer radiative lifetime according to (2). Therefore, introduction of PBBTDPP not only widens the absorption spectra range but also improves the efficiency of emission. 

## 4. Conclusion 

The stable ground structures of PBDPP, PBTDPP, and PBBTDPP have been optimized with density functional theory, and the energy levels, the electron density distribution, ionization potentials, and electron affinities have also been estimated in current calculations. The results show that the degree of coplanarity is increased from PBDPP, PBTDPP to PBBTDPP; for PBBTDPP it has the lowest IP and highest EA values, indicating that the well electron and hole transporting abilities among three oligomers. The absorption spectra of PBDPP and PBTDPP are mainly on the range of visible and ultraviolet light, while absorption spectra of PBBTDPP along with the introduction of the BBT unit make red-shifted to the infrared region. 3D charge difference density was used to analyze the calculated states, which indicated that the S_1_ state of PBBTDPP is an intramolecular charge transfer state (ICT), and change of electron density has obvious difference with that of PBDPP and PBTDPP. For the calculated excited states of three polymers, DPP of PBDPP and PBTDPP takes the role of electron donor or acceptor, and the certain role has wavelength dependence; but for PBBTDPP with the BBT unit, the DPP takes the role of electronic donor for most excited states, and PBBTDPP does not display the dependence of excitation energy.

## Supplementary Material

Charge different density of PBDPP, PBTDPP and PBBTDPP for the calculated excited states, where red and green colors stand for electron and hole, respectively.

## Figures and Tables

**Table 1:** Selected optimized bond length and dihedral angles of three polymers in the ground and excited state.

	T-DPP	DPP-T	T-T	T-B^a^(BT^b^/BBT^c^)	B^a^(BT^b^/BBT^c^)-T	T-T	T-DPP	DPP-T	T-T	T-B^a^(BT^b^/BBT^c^)	B^a^(BT^b^/BBT^c^)-T
Ground-state											
Band length^a^	1.44414	1.43808	1.44380	1.46121	1.46112	1.44365	1.43831	1.43780	1.44334	1.46211	1.46502
Band length^b^	1.44410	1.43750	1.44256	1.45332	1.45104	1.44133	1.43785	1.43708	1.44222	1.45462	1.45665
Band length^c^	1.44345	1.43563	1.43655	1.43691	1.43639	1.43502	1.43383	1.43406	1.43610	1.43885	1.44623
Inter-intra^a^	**18.96641**	17.96440	**−**9.94571	**21.51214**	20.40410	**−**9.96365	**13.76026**	**−**11.22646	0.78283	23.92343	**−**24.31071
Inter-intra^b^	**19.37309**	16.89443	**−**7.72928	**−1.82834**	**−**1.24510	**−**1.02007	**11.78848**	**−**13.89113	5.17861	**−**2.95138	**−**0.50836
Inter-intra^c^	**18.69428**	15.32628	**−**7.11406	**−0.27066**	**−**0.54157	**−**4.32656	**10.44882**	**−**12.26616	3.06632	**−**0.08805	**−**0.00809

Excited state											
Band length^a^	1.44101	1.42714	1.43019	1.44328	1.44280	1.41882	1.41232	1.42029	1.43002	1.45603	1.46231
Band length^b^	1.44248	1.42891	1.43112	1.43891	1.44230	1.42360	1.41655	1.42289	1.43045	1.44937	1.45356
Band length^c^	1.44169	1.43092	1.43088	1.43029	1.43409	1.42869	1.42470	1.42672	1.43000	1.43681	1.44327
Interintra^a^	**18.09190**	12.78461	**−**5.06364	**1.39836**	0.69850	**−**0.67672	5.70595	**−**8.55192	**−**0.66290	16.30034	**−**22.33211
Interintra^b^	**18.40059**	13.89040	**−**4.15560	**−0.47006**	**−**1.05672	**−**0.86839	3.82800	**−**10.31825	2.70990	**−**0.32685	0.00412
Interintra^c^	**17.93294**	13.76864	**−**4.96383	**−0.08738**	**−**0.34190	**−**1.99011	8.32850	**−**10.32846	1.68974	**−**0.07462	**−**0.04274

^a,b,c^Stand for PBDPP, PBTDPP, and PBBTDPP, respectively.

**Table 2:** Calculated transition energies (eV, nm), CI coefficients, and oscillator strengths (*f*) for PBDPP, PBTDPP, and PBBTDPP.

	State	*E* (eV/nm)	CI coefficients	Strength (*f*)
PBDPP	1	2.1891 (566.37)	(0.61429) H→L	2.7654
	2	2.4465 (506.77)	(0.52406) H − 1→L	0.5734
	3	3.0869 (401.64)	(0.38489) H − 2→L (0.37420) H→L + 1	0.1879
	4	3.3007 (375.63)	(0.37551) H − 3→L + 1	1.4478
	5	3.4302 (361.45)	(0.31621) H→L + 2	0.0079
	6	3.5060 (353.63)	(0.35875) H − 3→L + 1	0.1306
	7	3.6010 (344.30)	(0.55590) H − 6→L	0.0079
	8	3.6297 (341.58)	(0.50177) H − 8→L + 1	0.0109
	9	3.6501 (339.68)	(0.29316) H − 8→L + 1 (0.25410) H − 1→L + 3	0.0337
	10	3.7491 (330.70)	(0.32060) H→L + 1	0.0045

PBTDPP	1	1.9737 (628.18)	(0.55350) H→L	3.7597
	2	2.2795 (543.90)	(0.41198) H − 1→L	0.0488
	3	2.5570 (484.88)	(0.29044) H→L + 2 (0.29044) H − 2→L	0.2844
	4	2.6907 (460.79)	(0.37669) H→L + 3	0.1304
	5	2.9725 (417.11)	(0.39870) H − 1→L	0.0016
	6	3.1739 (390.63)	(0.38543) H − 2→L	0.0019
	7	3.2606 (380.25)	(0.35660) H − 3→L	0.4317
	8	3.3159 (373.91)	(0.28866) H − 2→L + 2 (0.28368) H→L + 4	0.0234
	9	3.4327 (361.19)	(0.26754) H − 2→L + 3 (0.26754) H − 3→L + 2	0.0366
	10	3.4761 (356.68)	(0.27111) H − 3→L + 3	0.3190

PBBTDPP	1	1.1434 (1084.32)	(0.60142) H→L	2.3231
	2	1.3824 (896.88)	(0.44301) H→L + 1	0.2545
	3	1.8970 (653.57)	(0.52437) H − 1→L	0.0131
	4	2.0583 (602.36)	(0.43842) H→L + 1	0.1684
	5	2.1955 (564.73)	(0.45129) H − 2→L	0.4115
	6	2.3714 (522.83)	(0.53522) H→L + 2	0.5742
	7	2.4918 (497.57)	(0.39465) H→L + 3	0.4517
	8	2.5491 (486.39)	(0.48457) H − 1→L + 1	0.0376
	9	2.6544 (467.09)	(0.42196) H − 3→L	0.0241
	10	2.9483 (420.52)	(0.56096) H − 4→L	0.0009

**Table 3:** Fluorescence energies (eV, nm), main configurations, and radiative lifetime of PBDPP, PBTDPP, and PBBTDPP.

	*E* (eV/nm)	CI coefficients	Strength (*f*)	Δ*E* ^a^ (nm)	τ^b^
PBDPP	1.8759 (660.93)	(0.69058) H→L	3.1267	94.56	2.0904
PBTDPP	1.7114 (724.45)	(0.56855) H→L	3.7192	96.27	2.1114
PBBTDPP	1.0208 (1214.63)	(0.61494) H→L	2.2405	130.31	9.8515

^a^Energy difference between absorption and emission peaks; ^b^lifetime (ns).
