# Supplementary material for: Effect of Electronic Acceptor Segments on Photophysical Properties of Low-Band-Gap Ambipolar Polymers
Source: ScientificWorldJournal. 2013 Jan 10;2013:890215. doi: 10.1155/2013/890215 (PMC3556892; doi:10.1155/2013/890215)
Supplement: Supplementary file 1 — Charge different density of PBDPP, PBTDPP and PBBTDPP for the calculated excited states, where red and green colors stand for electron and hole, respectively. [file 890215.f1.doc]

**Supporting materials**
